# Amygdala and Emotion: The Bright Side of It

**DOI:** 10.3389/fnins.2016.00224

**Published:** 2016-05-24

**Authors:** Mathias Weymar, Lars Schwabe

**Affiliations:** ^1^Department of Biological and Clinical Psychology, University of GreifswaldGreifswald, Germany; ^2^Department of Cognitive Psychology, University of HamburgHamburg, Germany

**Keywords:** emotion, amygdala, fMRI, affective neuroscience, arousal

It has long been known that the amygdala, a bilateral structure from the medial temporal lobe, is related to emotion, particularly in processing of aversive information (e.g., LeDoux, 1996). However, accumulating evidence suggests that amygdala activation is also involved in processing pleasant information, as observed, for instance, in studies using reward-learning (e.g., Adolphs, 2010; Janak and Tye, 2015), episodic memory encoding (e.g., Hamann et al., 1999; Dolcos et al., 2004), pleasant scene or face perception (e.g., Sabatinelli et al., 2011) or mental imagery of pleasant experiences (e.g., Costa et al., 2010). In a broader sense, the amygdala has been discussed as a key structure triggering the organisms' survival circuit that is organized into distinct motivational systems, the appetitive and defensive motivation system (Lang and Bradley, 2010). These systems engage processes that facilitate attention allocation and perceptual processing (Anderson and Phelps, 2001; Vuilleumier, 2005; Schwabe et al., 2011), associated with prioritized memory storage (McGaugh, 2015), and initiate metabolic changes (arousal) in order to mobilize the organism for appropriate coping actions, such as approach or avoidance (e.g., Lang and Bradley, 2013). Based on animal models (see for overview Davis and Whalen, 2001; Davis and Lang, 2003), these primary functions are served by bundles of nuclei within the amygdala, which receive input from various sources, such as cortex and thalamus (sensory) or hippocampus and in turn project to regions that mediate various cognitive functions (e.g., vigilance, attention, memory) as well as other processes (e.g., autonomic and somatic) and together facilitate such survival actions.

The activation of both motivating systems by pleasant or unpleasant external and internal events can also vary by intensity (arousal), which is described as the strength of motivational mobilization (Russell, 2003; Lang and Bradley, 2013). A majority of human neuroscience studies, however, found that the amygdala is activated by emotionally arousing stimuli, regardless of whether they are pleasant or unpleasant (Sabatinelli et al., 2011; but see Costafreda et al., 2008; Lindquist et al., 2016 for slight valence-differences), suggesting that the amygdala's predominant role may be the detection of emotionally arousing cues and subsequent activation of the organisms' motivational circuitry. A new study by Bonnet et al. (2015) provides additional evidence for such an “arousal” view. In this fMRI study, BOLD activity was measured while participants viewed pleasant visual scenes that varied in emotional arousal. This simple, but elegant, approach ensured that the outcome was not confounded with valence differences. Notably, in addition to neural activation, reactivity of the autonomic nervous system (skin conductance response) was simultaneously assessed in the scanner environment, and subjective ratings were obtained 1 month after scene encoding. The authors found that when pictures were rated high in arousal, stronger activation of the amygdala and hypothalamus was observed, as well as increased autonomic reactivity, in comparison to pictures receiving low arousal ratings. These results fit nicely with previous data showing covariations between sympathetic skin conductance responses and rated arousal levels of affective scenes, irrespective of their valence (e.g., Lang et al., 1993; Bradley et al., 2001). The findings of Bonnet and colleagues are also in line with animal data (summarized in e.g., Davis and Whalen, 2001; Lang and Davis, 2006) showing that projections from the amygdala (central nucleus) to the lateral hypothalamus prompt strong sympathetic activation, in terms of tachycardia, blood pressure elevation, pupil dilation and, likewise, increase in skin conductance. Although the amygdala is sensitive to arousing stimuli, “intensity detection,” as suggested by Bonnet and colleagues, seems not to be the only role for this structure (e.g., Adolphs, 2010). Previous studies found that the amygdala is also responsive to novelty and prediction error (e.g., Hamann et al., 2002; Blackford et al., 2010; Roesch et al., 2010; Wendt et al., 2011). These findings rather suggest that the amygdala, in a broader view, is important to tag salient cues in our environment or to assign values to important cues. The functional significance might be to interrupt on-going mental activity and automatically direct attention toward this external signal, and initiate other processes to promote appropriate motivational actions.

Although the data by Bonnet et al. (2015) appear to suggest that there is a common brain system that represents both unpleasant and pleasant affect, this is still under debate (e.g., Lang and Bradley, 2010; Fernando et al., 2013; Janak and Tye, 2015; Lindquist et al., 2016). Some cell populations in the amygdala have been found to respond to both fear- and reward-related stimuli in rats (Shabel and Janak, 2009), which were also related to activation of the autonomic nervous system (e.g., blood pressure), substantiating the view that the amygdala encodes saliency and triggers sympathetic nervous system activation. However, other cell ensembles in the amygdala encode unpleasant and pleasant value in a distinct fashion (e.g., Paton et al., 2006; Namburi et al., 2015). Especially, pleasant cues (e.g., Sabatinelli et al., 2007; Costa et al., 2010; Janak and Tye, 2015) have been shown to activate a neural circuit, including the amygdala, medial prefrontal cortex and the nucleus accumbens, that contributes more to reward-seeking behavior (e.g., Russo and Nestler, 2013), suggesting that some reactions in neural circuits are selectively prompted by appetitive cues. Future research needs to address how the amygdala interacts with these parallel and shared micro- and functional networks in the brain supporting salience and valence to affect specific motivated behavior (Janak and Tye, 2015; Lindquist et al., 2016). This could also help to better explain drug addiction (Kauer and Malenka, 2007), mood disorders (Russo and Nestler, 2013) and obsessive-compulsive disorders (Wood and Ahmari, 2015), which may be not only related to abnormalities in the adaptive function of the reward system, as assumed.

## Author contributions

MW has drafted this commentary, LS provided critical revisions. Both authors approved the final version of the manuscript.

### Conflict of interest statement

The authors declare that the research was conducted in the absence of any commercial or financial relationships that could be construed as a potential conflict of interest.
